# Interrelationships Among Changes in Leptin, Insulin, Cortisol and Growth Hormone and Weight Status in Youth

**DOI:** 10.4274/jcrpe.v3i1.05

**Published:** 2011-02-23

**Authors:** Kristin S Ondrak, Robert G McMurray, Anthony C Hackney, Joanne S Harrell

**Affiliations:** 1 University of North Carolina at Chapel Hill, Department of Exercise and Sport Science, Chapel Hill, NC, USA; 2 University of North Carolina, School of Nursing, Chapel Hill, NC, USA Address for; +1 919 609 04 18kristin.ondrak@gmail.comUniversity of North Carolina at Chapel Hill, Department of Exercise and Sport Science, Chapel Hill, NC, USA

**Keywords:** Weight change, leptin, cortisol, insulin, growth hormone, adoloscent, children

## Abstract

**Objective:** While acute alterations in leptin, insulin, cortisol and growth hormone (GH) levels have been reported in children following weight change interventions, little is known about natural hormonal changes as children grow and how these changes are affected by

unprovoked weight status changes. The purpose of this investigation was to compare changes in leptin, insulin, cortisol and GH levels in youth who maintained their weight status vs. those who moved from normal weight to overweight or vice versa.

**Methods:** Data were collected from 120 youth at baseline (9.8±1.0 years) and two years later. Participants were selected from a larger cohort to represent all scenarios of weight status: normal weight [>5^th^ and <85^th^ body mass index (BMI) percentile] at both time points (NN), overweight (≥85^th^ BMI percentile) at both time points (OO), normal weight status who changed to overweight (NO) and overweight status which changed to normal weight (ON). Hormonal concentrations were measured from fasting venous blood.

**Results:** In youth who changed their weight status, there were significant associations (p<0.05) between changes in BMI percentile and changes in leptin, insulin and cortisol (partial R^2^= 0.35, 0.13 and 0.11, respectively), after accounting for race, sex and changes in pubertal status and aerobic power. Our key findings were that youth who became overweight (NO) showed greater changes for leptin (+205% vs. -21%) and cortisol (-33% vs. +13%), p<0.05 than those who reverted from overweight to normal weight (ON).

**Conclusion:** Natural changes in weight status in youth show a relationship with changes in leptin, insulin and cortisol levels and the hormonal changes appear to be more sensitive to increases, rather than reductions, in weight status.

**Conflict of interest:**None declared.

## INTRODUCTION

Resting levels of several metabolic hormones are affected by weight status. Cross-sectional studies have shown that fasting leptin, insulin and cortisol are higher in overweight compared to normal weight children and adolescents (1,2,3,4,5,6), while growth hormone (GH) levels are lower (2). These differences may help to explain why overweight and obese children are at increased risk for future lifestyle diseases. While these studies highlight cross-sectional differences in the above-mentioned hormones, much less is known about the normal progression of these hormonal changes over time. Of the few longitudinal studies conducted on youth pertaining to these relationships, most have involved interventions aimed at reducing body weight or improving body composition. For example, researchers have reported reductions in insulin resistance (HOMA-IR) and leptin following substantial weight loss and increases in these hormones without substantial weight loss (7). Similar associations were noted for cortisol and insulin in another study by the same investigators, where significant reductions were found only in participants who lost substantial weight (8). Only one study of change in weight status and concomitant change in GH in youth was found, and these authors reported increased GH in prepubertal children following a weight loss program (9). Taken together, these relatively short-term studies demonstrate trends for decreased insulin, leptin and cortisol and increased GH following acute reductions in weight status. However, we know little about the changes in these hormones that accompany alterations in body or fat mass over time, as the youth age.

In this investigation, which we believe is the first, we describe the changes that occur over a two-year study period in leptin, insulin, cortisol and GH levels in normal 

and overweight youth and in youth who changed their weight status by either becoming overweight or normalizing their weight.This approach allowed us to compare values of several key metabolic hormones in youth who followed a normal pattern of weight gain to those who deviated from the normal  pattern by changing their weight status. Finally, this investigation is highly unique in that it does not involve any type of intervention and instead, describes natural changes in weight status and leptin, insulin, cortisol and GH levels in children of pubertal ages.

## METHODS

Participants were selected from the Cardiovascular Health in Children III (CHIC) study (J.S. Harrell, who was the PI of the CHIC study and credit was due). A total of 120 children of pubertal ages took part in the current study and the data were obtained by trained research assistants at the schools at baseline and two years later (follow-up). The youth represent all scenarios of weight status over the two-year period and were analyzed in four groups: normal weight group [>5^th^ and <85^th^ body mass index (BMI)  percentile for age and sex] at both time points (NN); overweight group (≥85^th^ BMI percentile for age and sex) at both time points (OO);  the group with normal weight at baseline and overweight at follow-up (NO); and the group overweight at baseline  and normal weight at follow-up (ON). Complete details of participant selection, matching and weight status scenarios can be found in Ondrak et al (5) (Table 1). This study did not involve any weight change 

intervention, so changes in weight status were assumed to occur as a result of natural processes or changes in behavior. Participants in the NN and OO groups represent what we would expect for a  normal growth pattern in youth who were of normal weight or overweight at both time points, respectively. Youth in the NO group provide valuable information about what happens when youth veer away from a normal growth pattern and become overweight, while the reverse is true for the ON group. The participants signed assent forms and their parent(s) signed informed consent forms prior to data  collection. All procedures were approved by the Institutional Review Board at the University of North Carolina at Chapel Hill.

Body weight and height were measured using an electronic scale (Model 2101KL, Healthometer Medical, Bridgewater, IL) and a stadiometer (Perspective Enterprises, Portage, MI). These measurements were taken with the participant barefoot and excess clothing removed (and pockets emptied) and were recorded to the nearest 0.1 kg and 0.1 cm The measurements were repeated a second time and the two measurements were averaged and used to calculate BMI. Research assistants measured skinfold thickness at the triceps and subscapular sites, using procedures set forth by the National Health and Nutrition Examination Survey (1974) (10). Measurements were taken in triplicate using Lange calipers (Cambridge Scientific Instruments, Cambridge, MD) and means calculated for each site. Percent body fat was calculated using equations specific to each sex, pubertal stage and race (11) and these values were used to estimate fat mass and fat-free mass (ffm).

In addition, pubertal development and aerobic power were assessed since they have been shown to influence insulin levels (12,13). Pubertal development was self-assessed via a questionnaire (14), while aerobic power was predicted using a submaximal test performed on the cycle ergometer, the PWC_195_ (15). We chose to express aerobic power in mL/kg_ffm_/min units in order to remove the influence of fat mass, and focus on the oxygen uptake of ffm since it is most related to glucose uptake. The following equation was used to calculate aerobic power in mL/kg_ffm_/min.

A blood sample was obtained by standard venipuncture between 7 and 9 am, following a verified 12 hour fast. The blood was centrifuged at 4^0^C, the plasma separated and stored at -80^0^C until analysis. Insulin, leptin, cortisol and GH levels were measured in duplicate from the stored plasma using radioimmunoassay (RIA) procedures (insulin and leptin: LINCO Research, St. Charles, MO; GH: MP Biomedicals, Costa Mesa, CA). The lowest detectable limits of these assays were 2 mU/mL, 0.5 ng/mL, and 0.31 ng/mL, respectively. Cortisol was measured using solid-phase, single-antibody RIA kits, with a lowest detectable limit of 0.2 mg/dL (Diagnostic Products Corporation, Los Angeles, CA). The mean coefficients of variation for the leptin, insulin, GH, and cortisol assays were 8.1%, 8.0%, 7.2%, and 6.4%, respectively.

**Statistical Analysis**

In order to identify group differences among participants at baseline, a one-way ANOVA was completed, comparing age, pubertal stage, height, weight, BMI, BMI percentile, percent fat,VO2/kg_ffm_, insulin, leptin, cortisol, and GH. Likewise, a Mantel-Haenszel chi-square was computed to reveal group differences at baseline for race and sex. To quantify the amount of change in leptin, insulin, cortisol and GH over the two-year study period, change scores 

(follow-up - baseline) were calculated for each participant. Changes in pubertal status were quantified as a dichotomous variable in subsequent analyses (no change or change). In females, change in pubertal stage was defined as moving from a pubertal stage of 1, 2 or 3 at baseline  to 4 or 5 at follow-up; for males change was defined  as moving from stages 1 or 2 at baseline to 3, 4 or 5 at follow-up.

Analyses of covariance (ANCOVA) were conducted for each hormone to determine weight group differences in the amount of change after controlling for race, sex, and the hormone values at baseline. Separate ANCOVAs  were computed for each of the following weight group comparisons: NO vs. ON, NN vs. NO and OO vs. ON. We included race as a covariate in the group comparisons, because there was an unequal distribution of races in each group. Sex was also  a covariate as it is known to influence resting levels of leptin in youth (16, 17). Additionally, initial examination of the data revealed that the majority of participants with elevated GH levels were African American females, further supporting the need for race and sex as covariates in our analyses.

Multiple regression analyses using the stepwise selection were performed to determine the association among changes in each of the hormones for participants in the NO and ON groups with changes in BMI percentile, controlling for race, sex and changes in pubertal status and VO2/kg_ffm_. We controlled for these variables since they are known to influence insulin in youth (18, 19). Collinearity diagnostics were calculated to check for collinearity among the independent variables in the regression models. According to the recommendations of Belsley et al (20), variables would be considered collinear if they had a high condition index (>30) and at least two variance proportions that are >0.5. Using these criteria, no variables were collinear. While the inclusion of only the NO and ON groups reduced our statistical power for the regression models, we chose to focus our analyses solely on the participants who changed their weight status. All statistical analyses were completed with SAS Statistical Software, Version 9.1(Cary, NC) and the a level was equal to 0.05.

**(Table 1):**
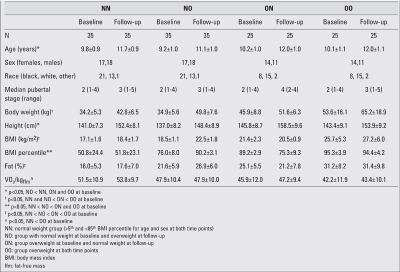
Participant characteristics (mean ± standard deviation), presented by weight status group at baseline and follow-up

## RESULTS

The participant characteristics at baseline and follow-up are shown in Table 1. In general, height, body mass and pubertal development increased over the two-year period, as expected. At baseline, participants in the OO and ON groups had a larger mean BMI percentile than those in the NN and NO groups. There were group differences in race, as the NN and NO groups had more black participants than the ON and OO groups. Participants in the NO group 

were younger than participants in all other groups by approximately one year (p<0.05). Finally, the OO group had higher mean insulin and leptin values at baseline, compared to all other groups.

**Weight Groups Comparisons**

Both the NO and ON groups underwent a 14-unit change in BMI percentile, albeit in opposite directions (Table 1). The NN and OO groups both stayed extremely stable, with BMI percentile changes of one point or less over the two years. There were significant differences between the NO and ON groups for changes in leptin and cortisol (p<0.001). As shown in Table 2, there was a large increase in mean leptin levels in the NO group (+205%), and a slight decrease for the ON group (-21%). For cortisol, the ON group increased slightly (+13%), while the NO group decreased by a larger magnitude (-33%). Although not statistically significant, insulin and GH levels increased for the NO group (+26% and 53%, respectively) and decreased in the ON group (-6% and -21%, respectively). 

To describe the changes associated with becoming overweight, we compared participants in the NO group to those in the NN group. There were significant differences for insulin and leptin, as the NO groups had a greater increase compared to changes in the NN groups (Table 2). Similarly, the NO group had a larger decline in cortisol, compared to the NN group. Finally, when comparing participants who normalized their weight (ON) to those who remained overweight (OO), significant group differences were found for leptin as the OO group increased markedly, while the ON group declined. Finally, the OO group had greater reductions in insulin compared to the ON group, p<0.05.

**Multiple Regression Models**

**Figure 1:**
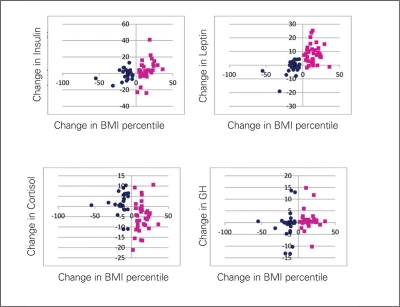
Scatterplots of changes in insulin, leptin, cortisol and GH and change in BMI percentile over two years for youth who increased (NO) or decreased (ON) their weight status NO: normal to overweight ON: overweight to normal weight

The results of the multiple regression analyses for the youth who underwent weight status change (NO and ON groups) showed significant partial correlations between changes in BMI percentile and changes in insulin (R^2^=0.13), leptin (R^2^=0.35) and cortisol (R^2^=0.11) levels, after adjusting for changes in pubertal status, VO2/kgffm and race and sex Table 3 and Figure 1). Despite the small total R^2^, change in BMI percentile accounted for the 

greatest amount of variance in changes in leptin, insulin and cortisol. A graphical representation of the relationship between changes in BMI percentile and changes leptin, insulin, cortisol and growth hormone can be found in Figure 1. This figure shows that the strongest relationships are between changes in BMI percentile and leptin, followed by changes in insulin and lastly, an inverse relationship with changes in cortisol.

**Table 1:**
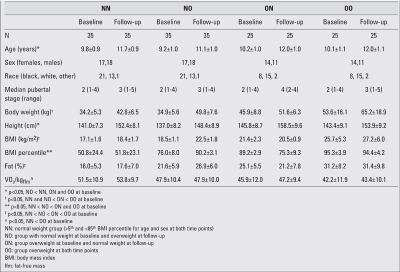
Participant characteristics (mean ± standard deviation), presented by weight status group at baseline and follow-up

**Table 2:**
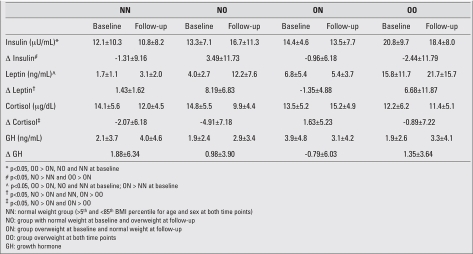
Insulin, leptin, cortisol and GH at baseline and follow-up, and changes in these hormones (mean ± standard deviation), presented by weight status group

**Table 3:**
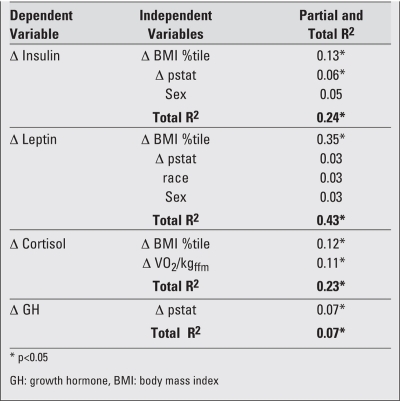
Multiple regression models of changes in insulin, leptin, cortisol and GH with respect to changes in BMI percentile, pubertal status, VO2/kgffm, as well as sex and race for participants who underwent change in weight status

## DISCUSSION

To our knowledge, this investigation is the first to describe the longitudinal relationships among natural changes occurring over time in weight status and changes in leptin, insulin, cortisol, and GH. A major finding is that changes in BMI percentile were related to changes in leptin, insulin and cortisol, even after controlling for factors known to influence these variables. Furthermore, our data suggested youth who became overweight over the study period had larger changes in these hormones than overweight youth who reverted to normal weight. When all hormonal relationships were considered, the strongest association was between change in BMI percentile and change in leptin Table 3. Likewise, the largest differences were found for leptin (NO=205% increase vs. ON=21% decrease). Taken together, these findings suggest that of the four hormones analyzed, changes in leptin are the most sensitive to changes in weight status (21, 22). The trends in leptin change of the ON and OO groups are similar to findings reported by Reinehr et al (7). Other studies have reported larger reductions in leptin in response to weight loss programs, which may be attributable to the greater starting values for leptin and percent body fat or BMI in their participants (23,24,25). Additionally, the relatively small change in leptin in the ON group in our study may be due to our classification of weight status. Our ON participants were only moderately overweight at baseline (mean of 89th BMI percentile) and they were at the very high end of the normal weight range (˜75^th^ BMI percentile) at follow-up. If these participants had started or ended with a higher or lower BMI percentile, respectively, greater changes in leptin, and all other hormones, may have been observed. Finally, it is important to point out that changes in weight status in our investigation were not invoked by an intervention per se, and they occurred as a result of natural processes over a two-year period. Thus, the related changes in hormones may differ markedly from studies that used a weight change intervention approach.

The large increase in leptin in the NO groups may be explained by the 79% increase in fat mass from baseline to follow-up, since leptin is released from adipocytes (21). Researchers have proposed a theoretical model of the relationship between insulin, leptin and adipose tissue, termed the “adipo-insular” axis, which may help to explain our findings (26). In this axis, insulin is thought to increase the release of leptin from adipose tissue, while leptin decreases the release of insulin. The smaller relative change in insulin in the current investigation may be partially explained by an inhibition of its release by increased leptin. Viewed collectively, our findings support the concept of an “adipo-insular” axis and an association between changes in leptin and insulin in children of pubertal ages.

The inverse association between changes in BMI percentile and cortisol was significant, but in the opposite direction of what we expected from previous research. These findings are somewhat puzzling, considering that the group means for cortisol were similar to previous reports for both normal (2,8) and overweight youth (2,8,27). In contrast to our findings, Reinher and Andler (8), using obese, insulin resistant subjects, reported reductions in cortisol after a one-year weight reduction program. Their results may have differed from our investigation due to the weight reduction intervention conducted in their study, leading to alterations in diet and exercise, or to the greater magnitude of weight change noted in the subjects. Regrettably, while the actual change in weight or fat mass was not reported in their investigation, the mean weight change was a reduction of approximately 3.3 BMI units, whereas the youth in our ON group experienced a BMI reduction of only 0.9 kg/m^2^. Other factors that may explain why our groups did not follow expected trends include the small absolute changes in cortisol with large standard deviations, and the within day variation in cortisol due to its circadian pattern of release (28). However, we obtained our samples between 7-9 am in a standardized fashion in an attempt to minimize this influence.

The change in GH did not differ among any of the weight status groups (p>0.05), and the magnitude of change was quite small. Notably, the NO group increased slightly, while the ON group decreased by a similar amount over the two-year study period. These results have not been reported previously and may be attributable to the low mean GH values and the large standard deviations in youth in this age group. These factors make it difficult to detect changes, which may have occurred specifically in response to weight status change. Also, our GH values were slightly lower than those previously reported for children of normal weight (29,30) and slightly higher than reported values for obese youth (9,29). Perhaps, if we had been able to take serial samples we may have been able to take into consideration the pulsatile nature of GH release, which may have provided more reliable results.

Athough a significant, inverse relationship between aerobic power per kilogram lean body mass and insulin was reported in a previous study (18), we failed to detect an association among changes in VO2/kg_ffm_ and changes in insulin, leptin or GH. These results suggest that fat mass may be a more critical component influencing these hormonal changes than fat-free mass. Additionally, our results may have differed from the investigation reported by Allen et al (18), as their participants were obese (BMI >95^th^ percentile) and the study was assessing cross-sectional relationships. Finally, the two-year changes in VO2/kg_ffm_ in our study were relatively small (3%), which may explain why we did not detect associations in their change scores in relation to the hormonal changes.

There were a number of strengths and weaknesses in this investigation. Our design was highly unique as it examined normal changes in weight status, as opposed to those caused by an intervention. This design improves the generalizability of our results, as the vast majority of children are not involved in weight change intervention programs. By studying those who increased, decreased or maintained their weight status, we have representation from all possible scenarios of weight status change in youth of this age group. These distinctions are important when using longitudinal designs in youth, since they are likely to gain weight as they grow, but their BMI percentile may actually be decreasing if their height is increasing at a faster rate than their body weight. Additionally, the classification of normal weight and overweight used in this study is commonly used by practitioners and researchers to delineate levels of disease risk and to make health recommendations. Examination of the NO and ON groups in particular allows us to describe what happens when youth move from a recommended weight status to one associated with increased risk, or vice versa and importantly, fail to track from year to year. 

A limitation associated with this investigation is the variability in hormone levels. Leptin (31) and GH (9, 32) are released in a pulsatile fashion and cortisol is also pulsatile and exhibits a circadian rhythm (33). By only obtaining one blood sample at each time point, it is difficult to determine if the hormone levels varied due to the time of day or other factors such as weight status. In an effort to reduce the effect of diurnal variability on hormone levels, we standardized the time of day during which blood samples were taken. However, future investigations in this area may consider repeated sampling of hormones throughout the day (34). Another limitation of our study is the variation in race and pubertal status among participants. We attempted to account for these differences by matching participants in the NO and ON groups to those in the NN and OO groups by their race and pubertal status at baseline; however, race may still have influenced our hormonal results. It is also known that children may progress through puberty at different rates and this is another potential limitation of this study. Nevertheless, the influence of pubertal stage change was likely to be small, as 83% of the participants had either no change or an advance of only one pubertal stage over the two-year study period.

In conclusion, in a sample of children of pubertal ages who increased or decreased their weight status over a two-year period, changes in BMI percentile were positively related to changes in leptin and insulin levels, inversely related to changes in cortisol and not related to changes in GH levels. Youth who became overweight showed larger changes in leptin and cortisol than overweight youth who reverted to normal weight. The results also 

suggest that gain in weight status has more of an impact on hormonal changes than reduction in weight status. It is possible that the favorable changes associated with normal reductions in weight status take longer to accrue than the negative effects of increase in weight status.

**Table 3:**
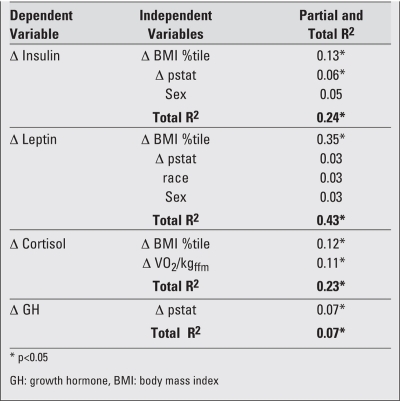
Multiple regression models of changes in insulin, leptin, cortisol and GH with respect to changes in BMI percentile, pubertal status, VO2/kgffm, as well as sex and race for participants who underwent change in weight status

## ACKNOWLEDGEMENT

Funding for this study was provided by NINR #NR01-1837.
